# Effects of repeated menstrual pain on empathic neural responses in women with primary dysmenorrhea across the menstrual cycle

**DOI:** 10.1002/hbm.25226

**Published:** 2020-10-08

**Authors:** Chen Wang, Yang Liu, Wanghuan Dun, Tian Zhang, Jing Yang, Ke Wang, Junya Mu, Ming Zhang, Jixin Liu

**Affiliations:** ^1^ Center for Brain Imaging, School of Life Science and Technology Xidian University Xi'an China; ^2^ Engineering Research Center of Molecular & Neuroimaging，Ministry of Education Xi'an China; ^3^ Department of Medical Imaging, First Affiliated Hospital of Xi'an Jiaotong University Xi'an China

**Keywords:** anterior insula, functional magnetic resonance imaging, pain empathy, pain perception, primary dysmenorrhea

## Abstract

Primary dysmenorrhea (PDM) is cyclic menstrual pain in the absence of pelvic anomalies, and it is thought to be a sex‐hormone related disorder. Existing study has focused on the effects of menstrual cramps on brain function and structure, ignoring the psychological changes associated with menstrual pain. Here we examined whether pain empathy in PDM differs from healthy controls (HC) using task‐based functional magnetic resonance imaging (fMRI). Fifty‐seven PDM women and 53 matched HC were recruited, and data were collected at the luteal and menstruation phases, respectively. During fMRI scans, participants viewed pictures displaying exposure to painful situations and pictures without any pain cues and assessed the level of pain experienced by the person in the picture. Regarding the main effect of the pain pictures, our results showed that compared to viewing neutral pictures, viewing pain pictures caused significantly higher activation in the anterior insula (AI), anterior cingulate cortex, and the left inferior parietal lobule; and only the right AI exhibited a significant interaction effect (group × picture). Post‐hoc analyses confirmed that, relative to neutral pictures, the right AI failed to be activated in PDM women viewing painsss pictures. Additionally, there was no significant interaction effect between the luteal and menstruation phases. It suggests that intermittent pain can lead to abnormal empathy in PDM women, which does not vary with the pain or pain‐free phase. Our study may deepen the understanding of the relationship between recurrent spontaneous pain and empathy in a clinical disorder characterized by cyclic episodes of pain.

## Graphical abstract

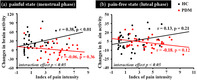

## 1 INTRODUCTION

Pain empathy is a complex form of psychological inference in which another person's pain is recognized and understood through a combination of observation, memory, knowledge, and reasoning (Ickes, 1993; Jackson, Meltzoff, & Jean, 2005; Martinotti, Di Nicola, Tedeschi, Cundari, & Janiri, 2009). Neuroimaging studies using functional magnetic resonance imaging (fMRI) have consistently shown that a core network consisting of the bilateral anterior insula (AI) cortex and anterior cingulate cortex (ACC), plays an important role in pain empathy (Singer et al., 2004). Results of meta‐analyses pointed out that the somatosensory cortex was also a hub node in pain empathy network (Jauniaux, Khatibi, Rainville, & Jackson, 2019; Lamm, Decety, & Singer, 2011). Furthermore, brain regions that are closely associated with pain empathy are reported to frequently experience plastic morphological changes in chronic pain patients (A. V. Apkarian, Hashmi, & Baliki, 2011; Deneen, Zhao, & Liu, 2019; Li Hu & Iannetti, 2016; L. Hu & Iannetti, 2019; M Catherine Bushnell, Marta, & Low, 2013; Von Deneen et al., 2019). Given that brain regions with abnormal structures elicited by chronic pain partially overlap with brain areas involving pain empathy, there is a strong possibility of abnormal pain empathy in chronic pain patients. However, it remains unclear what influence prior pain experience or prolonged nociceptive input in clinical populations has on empathy for pain.

Primary dysmenorrhea (PDM) is classified as a chronic pelvic pain syndrome. In female adolescents, it is a very common cyclic menstrual pain without pelvic pathology (Low et al., 2018; L. Yang et al., 2019). Functional neuroimaging studies revealed that short‐lasting cyclic menstrual pain leads to alteration of the gray matter volumes in some brain regions, including the AI, cingulate cortex, and hypothalamus (Low et al., 2018). Women with PDM are often accompanied by physiological disorders and negative emotions (Low et al., 2018; L. Yang et al., 2019), which may make it difficult for people to use emotional‐communicative information to infer pain in others (Preis, Schmidt‐Samoa, Dechent, & Kroener‐Herwig, 2013). Hence, further investigations into the mechanisms of pain empathy in PDM are warranted, to potentially eliminate psychological distress and improve the quality of life in women with PDM.

Various authors have argued that a person's capacity for empathy varies with the current state (Baron‐Cohen, Richler, Bisarya, Gurunathan, & Wheelwright, 2003; Batson & Shaw, 1991; Eisenberg & Strayer, 1990). For example, when individuals are angry or depressed, they may temporarily be unable to empathize with other people's pain (Baron‐Cohen et al., 2003; Meng, Chen, & Huang, 2010). People suffering from PDM with cyclic dysmenorrhea tend to experience a spontaneous pain‐free (periovulatory and luteal phase) and painful state (menstruation phase) within a menstrual cycle (Liu et al., 2017; Tu et al., 2013). Low and his colleagues reported that PDM showed differences in cerebral glucose metabolism and spontaneous functional activity between the periovulatory and menstruation phases (Low et al., 2018). They indicated that PDM can serve as a unique model for studying trait‐ and rapid state‐related brain changes from pain. Based on the above findings, PDM may also be a good clinical model for studying pain empathy in different pain states because of its natural painful and pain‐free phases.

In this study, we aimed to investigate how empathy‐related neural circuits in PDM subjects differ from those in healthy controls (HC), and we further examined whether or not there are differences in pain empathy between the painful and pain‐free phases of PDM. To validate this assumption, behavioral data assessing empathy‐related abilities were collected from 57 PDM and 53 age‐matched HC. Task‐based fMRI was used to measure the cerebral hemodynamic signals in response to pictures displaying people in potentially painful and pain‐free situations, during both the luteal and menstruation phases.

## 2 MATERIALS AND METHODS

This present study was conducted according to the Declaration of Helsinki and was approved by the Institutional Review Board of the First Affiliated Hospital of the Medical College in Xi'an Jiaotong University (L. Yang et al., 2019). All participants signed informed consent forms. The inclusion criteria for PDM individuals were as follows: (a) satisfy the diagnostic criteria for PDM, as per the American College of Obstetricians and Gynecologists; (b) had a regular menstrual cycle (27–32 days) in the last 6 months; (c) experience lower abdominal pain during menstruation, which affects everyday activities, but without any underlying pathologic change within or outside the uterus; and (d) their average cramping pain intensity in the last 6 months was rated >4 (0 = not at all, 10 = the worst pain). The PDM individuals were screened and diagnosed by a gynecologist from the Department of Obstetrics and Gynecology at the First Affiliated Hospital of the Medical College of Xi'an Jiaotong University (L. Yang et al., 2019). Inclusion criteria for HCs were as follows: (a) a regular menstrual cycle (27–32 days); and (b) no cramping pain or other symptoms during menstruation in the last 6 months.

Exclusion criteria for all participants were as follows: (1) organic pelvic lesions; (2) history of traumatic brain injury; (3) neurological disease or psychiatric disorder; (4) coexisting chronic pain conditions, such as irritable bowel syndrome, painful bladder syndrome, fibromyalgia, etc.; (5) ingestion of oral‐contraceptive drugs within the last 6 months; (6) immediate plans for pregnancy or a positive pregnancy test; (7) history of childbirth; and (8) any contraindications to MRI scans.

Fifty‐seven right‐handed PDM individuals and fifty‐three education‐ and age‐matched right‐handed HC individuals were recruited. All participants came from the local university and had been experiencing regular menstrual cycles during the previous six months. Each participant underwent two MRI scans during the experiment. The first scan (menstruation phase) was performed between the first and second day of the menstrual cycle, while the second scan (luteal phase) was performed 7–8 days after ovulation, or between the 21st and 22nd day of the menstrual cycle. Urine kits for luteinizing hormone were used to verify the exact time the female participants ovulated. Progesterone and estrogen levels were measured before the second scan to further confirm whether the participants were in the luteal phase. For PDM women, lower abdominal cramping pain was present in the menstruation phase (pain phase) but not in the luteal phase (pain‐free phase). Among these participants, 7 out of the 57 PDM individuals and 5 out of 53 HC individuals were excluded because of excessive head motion or fMRI data corruption. Another 6 and 4 individuals were excluded from the PDM and HC group, respectively, due to technical failures during pain rating, in response to painful and pain‐free pictures during the fMRI scan. And a further 2 and 4 individuals were excluded from the PDM and HC groups, respectively, due to them only having imaging and behavioral data for the pain‐free phase. Finally, 42 PDM participants and 40 HC participants were included in the subsequent analyses.

### 2.1 Visual stimuli

To elicit the participants' empathy for the pain of others, a series of pictures either related or unrelated to pain were used as experimental visual stimuli (Nicolas, Isabelle, & Roland, 2009; Schott, 2015). The picture stimuli used in this study were developed and validated by Jackson et al. (2005) (Jackson et al., 2005). Forty‐eight digital color pictures showing right hands and right feet in painful and pain‐free situations were presented. All of the scenarios in the pictures showed familiar events that can occur in daily life (Jackson et al., 2005; Moriguchi et al., 2007). There were various types of nociceptive stimuli in the pictures (e.g., mechanical, thermal, and stressful). The target persons in the pictures varied in gender and age (between 22 and 31 years old). To avoid biases in judgment caused by age and gender, the body parts involved in the pictures were smoothed (Moriguchi et al., 2007). The neutral pictures had the same background elements as the corresponding neutral pictures, except that the neutral pictures had no pain‐related elements (Jackson et al., 2005; Moriguchi et al., 2007). The target persons in the pictures were based on a gender‐balanced sample of 24 independent subjects. All pictures were edited to the same size and resolution (600 × 450 pixels).

### 2.2 Stimulation paradigm

**FIGURE 1:**
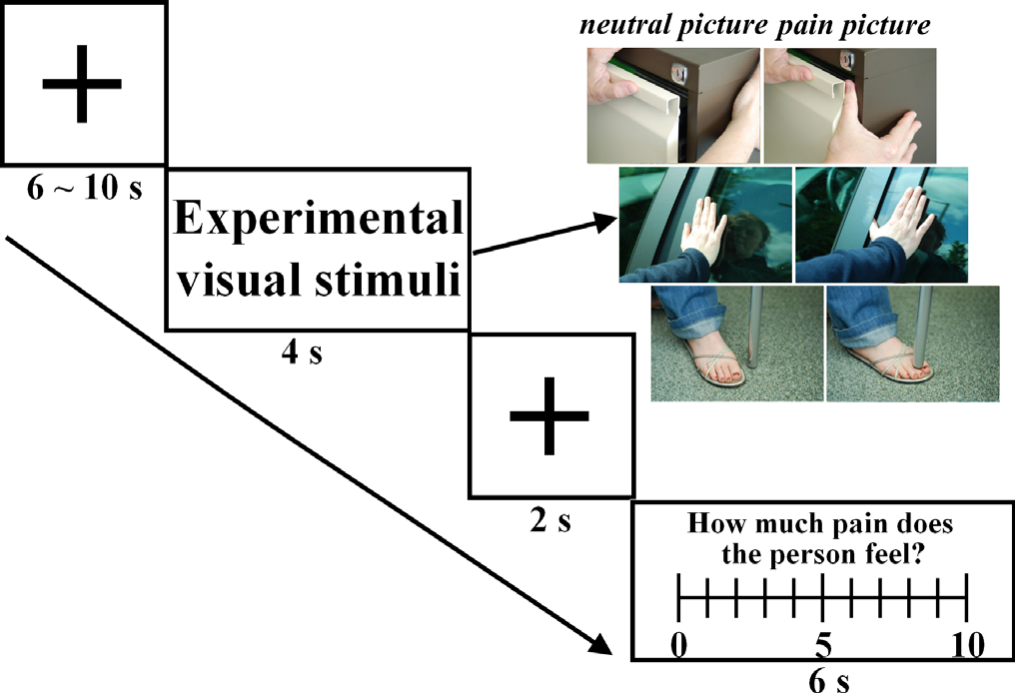
Stimulation paradigm

This present experiment consisted of two sessions. The stimulation paradigm used in each session was developed and validated by Preis et al. (2013) (Preis et al., 2013). Specifically, the participants underwent 24 trials grouped into 4 blocks (6 trials in each block). A trial consisted of a picture (4 seconds viewing a pain picture or neutral picture), followed by a fixation cross (2 seconds), and the pain rating (6 seconds) (Figure 1). The interval between the trials (black fixation cross on white background) was randomly varied between 6 and 10 seconds (mean duration = 8 seconds). After presentation of the pictures, the participants were instructed to imagine how the person in the picture feels (0 = no pain, 10 = the worst pain) (Preis et al., 2013).

### 2.3 Psychological assessment

A self‐rating anxiety scale (SAS) and self‐rating distress scale (SDS) were used to evaluate the participant's anxiety and depression levels (William W Zung, 1965; William W Zung, 1971). The visual analog scale (VAS) was used in the PDM and HC groups to assess their present experience of menstrual pain (0 = no pain, 10 = the worst pain) (Carlsson, 1983). The Short Form McGill Pain Questionnaire (SFMPQ) was used to evaluate how chronic pain influences participants' sensory, affective, and present feelings (Melzack, 1975, 1987). It consists of two subscales that measure the sensory and affective aspects of pain. Additionally, the present pain intensity (PPI) index of the standard McGill Pain questionnaire is also included in the SFMPQ (Melzack, 1975, 1987). Participants' empathetic ability was measured using a Chinese version of the Interpersonal Reactivity Index (IRI) (M. H. Davis, 1980; Zhang, Dong, & Wang, 2010). The IRI has the following four subscales: empathic concern, perspective taking, fantasy scale, and personal distress (M. H. Davis, 1980).

### 2.4 Scanning procedure

Before beginning the magnetic resonance scanning, participants completed the SAS, SDS, VAS, SFMPQ, and IRI. After the structural scan, two continuous task‐based fMRI scanning sessions were done, and the pictures were randomly presented (stimulation paradigm). After the presentation of each picture during each fMRI scanning session, participants were asked to rate the pain of the photographed target person on a comparable numeric rating scale.

### 2.5 Image acquisition

Scanning was done using a 3.0 T Signa GE scanner with an 8‐channel phased‐array head coil. For each subject, a high‐resolution structural image was acquired through a three‐dimensional MRI sequence with a voxel size of 1 mm × 1 mm × 1 mm, using an axial fast spoiled gradient recalled sequence with the following parameters: repetition time = 1900 ms, echo time = 2.6 ms, data matrix = 256 **×** 256, and field of view (FOV) = 256 **×** 256 mm.

The functional datasets were acquired using a T2*‐weighted echo‐planar imaging sequence. The parameters were as follows: repetition time = 2000 ms, echo time = 30 ms, data matrix = 64 × 64, voxel size = 3.75 mm × 3.75 mm × 4 mm, FOV = 240 mm × 240 mm, flip angle = 90°, and slices = 35 with no gap.

### 2.6 Data preprocessing

Functional MRI data preprocessing of a single subject was performed using the FEAT (functional magnetic resonance imaging expert analysis tool) toolbox in the Oxford Centre of Functional Magnetic Resonance Imaging of the Brain's (FMRIB) Software Library (FSL, version 5.0.9, see www.fmrib.ax.ac.uk/fsl) (Stephen Smith et al., 2001; Stephen M. Smith et al., 2004; M. W Woolrich, Ripley, Brady, & Smith, 2001; Mark W Woolrich et al., 2009). The first five volumes of each session were excluded to achieve the equilibrium of magnetization. The inter‐scan movements of the remaining functional images were eliminated using the Motion Correction FMRIB's Linear Image Registration Tool (Mark Jenkinson, Bannister, Brady, & Smith, 2002). The fMRI image was then spatially smoothed using an isotropic Gaussian kernel of 5 mm full‐width half‐maximum, and high‐pass filtering (cutoff period of 100 s) was applied to remove low‐frequency artifacts (Nicolas et al., 2009; M. W Woolrich et al., 2001). Spatial normalization was performed following a two‐stage process (Maleki et al., 2012). Firstly, using FLIRT (FMRIB's Linear Image Registration Tool), a low‐resolution fMRI image was linearly registered to the high‐resolution skull‐stripped structural image (Mark Jenkinson et al., 2002; Mark Jenkinson, Pechaud, & Smith, 2005; M. Jenkinson & Smith, 2001). The structural image was then normalized to the standard T1 Montreal Neurological Institute 152–2 mm space, using FMRIB's Nonlinear Image Registration Tool (Andersson, Jenkinson, & Smith, 2007; Mark Jenkinson et al., 2005; Stephen M. Smith, 2010). Finally, the registration parameters from combining the above two transformations were applied to the functional images (the statistical images were derived from the first‐level analyses), which were resampled to a 2 mm isotropic voxel size (Maleki et al., 2012; Nicolas et al., 2009).

### 2.7 First‐level analysis

The first‐level analysis of each subject was calculated using the FEAT toolbox (M. W Woolrich et al., 2001). The visual stimuli were modeled using a double‐gamma hemodynamic response function and its temporal derivatives (Hopfinger, Büchel, Holmes, & Friston, 2000; Woolrich, Behrens, & Smith, 2004). The models also included six head motion parameters (as confound explanatory variables) per session, in order to remove the effect of motion artifacts (Mark Jenkinson et al., 2002). Additionally, the motor‐related pain rating was also included in the model. Three contrasts of parameter estimates (COPE) were performed for each subject: P (Pain pictures ‐ Baseline), N (Neutral pictures ‐ Baseline), and PN (Pain pictures–Neutral pictures) (Nicolas et al., 2009).

### 2.8 Higher‐level analysis

The COPE from combining across sessions for each participant was calculated using the fixed effect higher‐level analysis in the FEAT toolbox (M. W Woolrich et al., 2001). In the following mixed‐effect analysis, a two‐way analysis of variance (ANOVA) based on regions of interest (ROI) was done using FLAME1 (FMRIB's Local Analysis of Mixed Effects) (Friston et al., 1998; Nicolas et al., 2009; Woolrich, Behrens, Beckmann, Mark, & Smith, 2004). Empathy‐related and pain‐related brain regions were chosen as the ROIs downloaded from the meta‐analytical database of NeuroSynth (www.neurosynth.org) which is a platform for the large‐scale, automated synthesis of fMRI data extracted from published articles (Yarkoni, Poldrack, Nichols, Van Essen, & Wager, 2011). We used the keywords “empathy” and “pain” in the search of the NeuroSynth repository. The resulting images (187 and 516 for empathy and pain, respectively) from 703 PubMed publications were mainly constrained to the bilateral AI, ACC, amygdala, precuneus, thalamus, and somatosensory areas (*p* < .01, FDR corrected).The following contrasts were performed using three COPE at the individual level: (a) the main contrast of group (HC vs PDM); (b) the main contrast of picture (P vs N); and (c) the interaction of picture and group (HC [P ‐ N] vs PDM [P ‐ N]) (Nicolas et al., 2009). Additionally, we also performed a three‐way repeated measures ANOVA with the between‐subject factors group (HC, PDM) and the within‐subject factors visual stimuli (pain pictures, neutral pictures) and state (painful, pain‐free). A permutation test (permutated 5,000 times) with threshold‐free cluster enhancement (TFCE) was utilized for multiple comparison corrections (T. E. Nichols & Holmes, 2002; S. M. Smith & Nichols, 2007; S. M. Smith & Nichols, 2009; Winkler, Ridgway, Webster, Smith, & Nichols, 2014). The statistical significance threshold was set to *p* < .05, using TFCE with a family‐wise error (FWE) correction for multiple comparison corrections (5,000 permutations) (Thomas E Nichols, 2012).

### 2.9 Correlational analyses

To further characterize whether the linear relationship between the change in hemodynamic signals induced by visual stimuli and the individual's index of pain intensity (reactivity to pain) differed between the two groups, analysis of group × covariate interaction was computed (Neter, Kutner, Nachtsheim, & Wasserman, 1996; Nicolas et al., 2009). The index of pain intensity was the pain intensity rating differences between the pain picture and neutral picture (Jackson et al., 2005). The value for contrast of parameter estimates of PN was extracted to represent a change in hemodynamic signals. Based on the above result, the Spearman's rho correlation was further used to measure the strength of association between the change in hemodynamic signals induced by visual stimuli and the individual's index of pain intensity (reactivity to pain) in each group.

## 3 RESULTS

### 3.1 Demographic and behavioral characteristics

**FIGURE 2:**
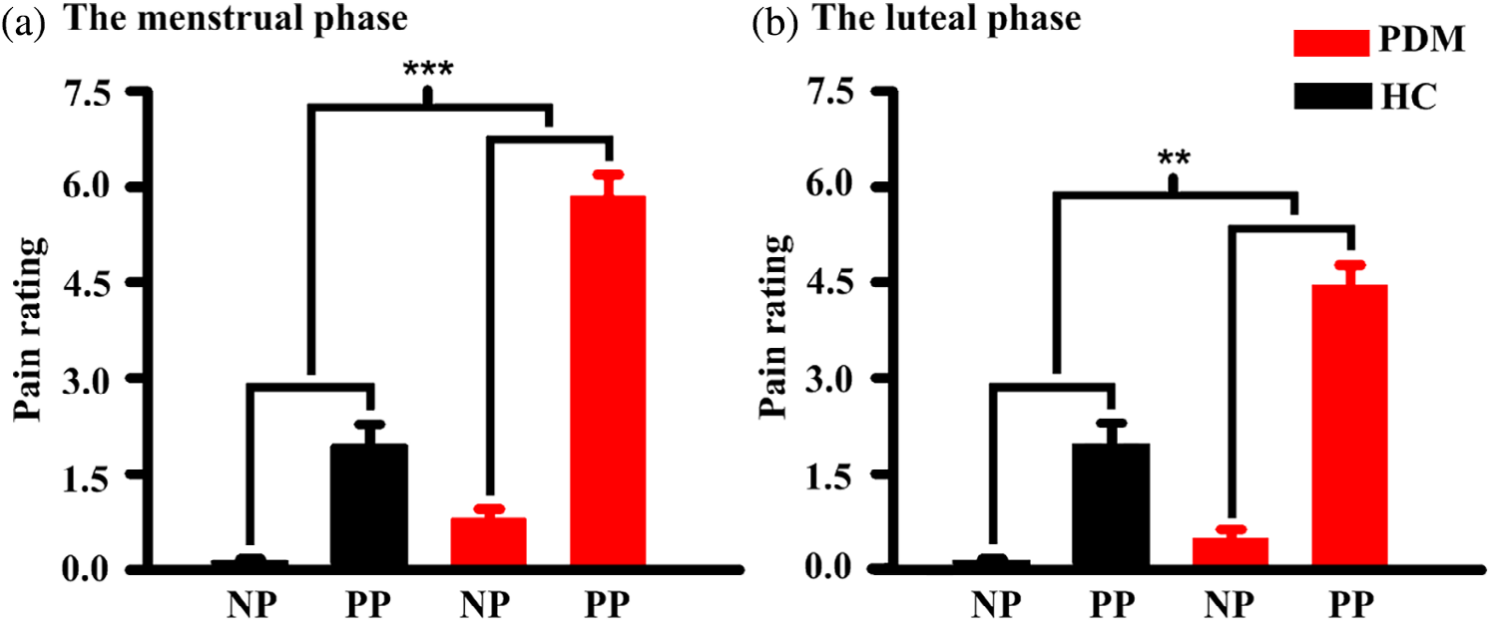
Between‐group differences in pain rating in response to visual stimuli. (a) Comparison of the two groups during the menstrual phase, for pain rating with different types of visual stimuli. (b) Comparison of the two groups during the luteal phase, for pain rating with different types of visual stimuli. The asterisks show that there was a significant interaction effect (group × picture) for the pain rating. *** *p* < .001, ** *p* < .01. HC, healthy controls; PDM, women with primary dysmenorrhea; NP, neutral pictures; PP, pain pictures

In this study, there were no significant differences between the PDM and HC groups (*p* > .05, Table 1) in any of the following factors: age, years of education, age at menarche, trait empathy assessed by the IRI, and average days of one menstrual cycle. All PDM individuals in our study had a long history of menstrual pain (10.7 ± 2.07 years), with duration of pain of 1–3 days in a menstrual cycle (2.05 ± 0.72 days). The group × picture mixed ANOVA on the pain rating revealed a main effect of group (*p < .001*), a main effect of picture (*p < .001*), and an interaction of time × group (*p < .001*) during the painful state (Figure 2(a)). The same results were observed during the pain‐free phase (Figure 2(b)). However, the group × picture × state three‐way repeated measures ANOVA on the pain rating did not reveal a significant interaction effect (*p* = .096). Compared with the HC group, the PDM group had significantly higher pain ratings for the pain pictures, during both painful and pain‐free states (*p* < .05, Table 1). In the SAS, there were significant differences for the painful state, but no differences were found for the pain‐free state. During the painful state, the SFMPQ, PPI, and VAS values for the PDM group were significantly higher than those for the HC group.

**TABLE 1:** Demographic and behavioral assessment

	Mean ± SE		
	HC (n = 40 )	PDM (n = 42)	*p* value
Age (years)	24.1 ± 0.25	24.3 ± 0.38	.65
Education (years)	17.4 ± 0.18	17.6 ± 0.23	.47
Cycles (days)	29.3 ± 0.27	29.2 ± 0.25	.75
Age at menarche (years)	12.4 ± 0.15	12.5 ± 0.15	.61
History of menstrual pain (years)		10.7 ± 0.32	
Duration of menstrual pain (days)		2.05 ± 0.11	
Luteal phase (pain‐free state)			
SAS	28.7 ± 1.12	31.6 ± 1.11	.07
SDS	31.1 ± 0.97	35.1 ± 1.31	.01
Fantasy	15.1 ± 0.65	15.2 ± 0.60	.86
Perspective taking	10.9 ± 0.56	10.6 ± 0.48	.66
Empathic concern	17.0 ± 0.48	17.2 ± 0.46	.73
Personal distress	7.6 ± 0.59	8.2 ± 0.56	.51
Pain rating for neutral pictures	0.2 ± 0.05	0.5 ± 0.12	.01
Pain rating for pain pictures	1.91 ± 0.31	4.4 ± 0.34	<.001
Menstrual phase (painful state)			
VAS	0.4 ± 0.15	4.2 ± 0.32	<.001
SAS	28.3 ± 1.14	34.2 ± 1.50	.003
SDS	30.2 ± 1.26	36.9 ± 1.67	.002
SFMPQ			
Sensation	0.8 ± 0.18	6.0 ± 0.86	<.001
Affective	0.9 ± 0.16	3.9 ± 0.45	<.001
PPI	0.4 ± 0.08	1.9 ± 0.15	<.001
Fantasy	14.5 ± 0.62	15.4 ± 0.66	.32
Perspective taking	11.0 ± 0.62	10.7 ± 0.58	.74
Empathic concern	16.3 ± 0.44	16.7 ± 0.49	.47
Personal distress	7.4 ± 0.58	8.0 ± 0.58	.48
Pain rating for neutral pictures	0.1 ± 0.04	0.7 ± 0.14	<.001
Pain rating for pain pictures	1.7 ± 0.30	5.2 ± 0.30	<.001

*Note:* The comparisons of subjects' basic information were performed between PDM and HC groups using two‐sample t test. *p<.05* was considered significant.

Abbreviations: HC, healthy controls; PDM, primary dysmenorrhea; PPI, the present pain intensity; SAS, self‐rating anxiety scale; SDS, self‐rating distress scale; SE, standard error; SFMPQ, the Short Form McGill Pain Questionnaire; VAS, visual analogue scale.

### 3.2 Between‐group differences in brain activity in response to visual stimuli during the menstrual phase

**FIGURE 3:**
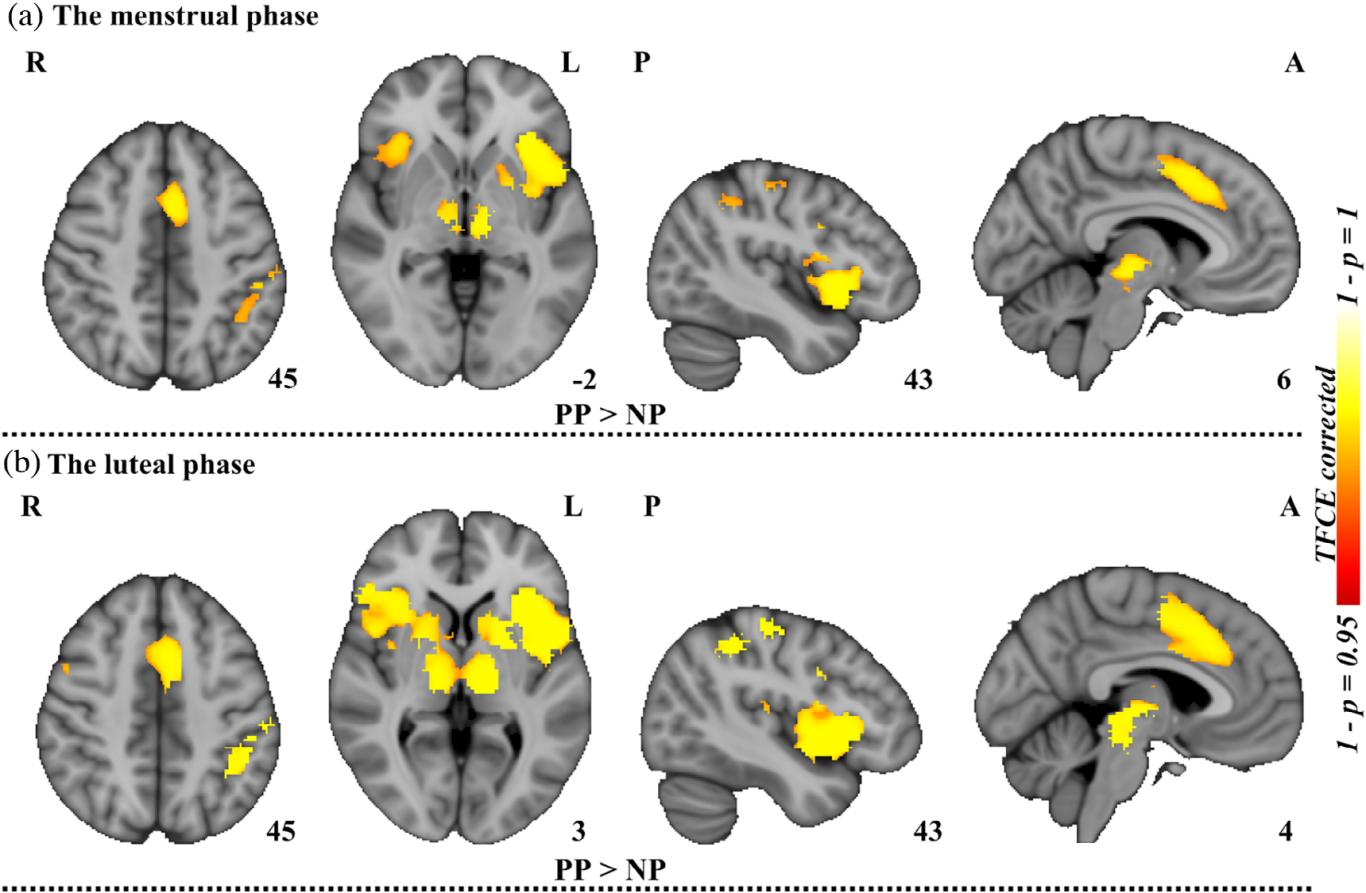
Between‐picture differences in brain activation. (a) Significant brain areas, from task activation comparisons conducted on viewing neutral pictures versus pain pictures, for all of the subjects during the menstrual phase. (b) Significant brain areas, from task activation comparisons conducted on viewing pain pictures versus neutral pictures, for all of the subjects during the luteal phase. Permutation test (permuted 5,000 times) with threshold‐free cluster enhancement (TFCE) was utilized for multiple comparison corrections. NP, neutral pictures; PP, pain pictures; L, left; R, right; A, anterior; P, posterior

During the painful state, the main effect of pictures (painful vs. neutral pictures) showed increased brain activities mainly in the bilateral AI, left MCC and left thalamus (*p* < 05, TFCE corrected, Figure 3(a) and Table 2). There were some additional activations in the left supramarginal gyrus, left rolandic operculum, left precentral gyrus, and the left supplementary motor area (Table 2).

**TABLE 2:** Results of main effect and interaction effect during the menstrual phase (*p < .05*, TFCE corrected based on the defined ROIs)

			Peak coordinate			
Region of activation	Side	Sizes	x	y	z	*t‐score*
The main effect of picture (PP vs NP)						
Insula	L	433	‐32	22	0	5.4531
Insula	R	169	36	24	‐4	4.0643
Thalamus	L	38	‐10	‐16	0	4.7605
Supramarginal gyrus	L	64	‐53	‐29	38	4.1346
Supplementary motor area	L	75	‐4	10	44	4.8142
Middle cingulate gyrus	L	114	‐6	13	44	4.5982
Inferior frontal gyrus,opercular part	L	168	‐55	15	29	3.7978
Precuneus	L	97	‐53	5	21	4.3458
Rolandic operculum	L	29	‐50	7	6	4.3882
The interaction effect (HC [PP ‐ NP] vs PDM [PP ‐ NP])						
Insula	R	57	34	25	‐4	3.9755

*Note:* The ROIs is defined as empathy‐related and pain brain‐related regions from the meta‐analytical database of NeuroSynth. Peak coordinates refer to the MNI space.

Abbreviations: L, left hemisphere; MNI, Montreal Neurological Institute; NP, neutral pictures; PP, pain pictures; R, right hemisphere; ROIs, regions of interest.

**FIGURE 4:**
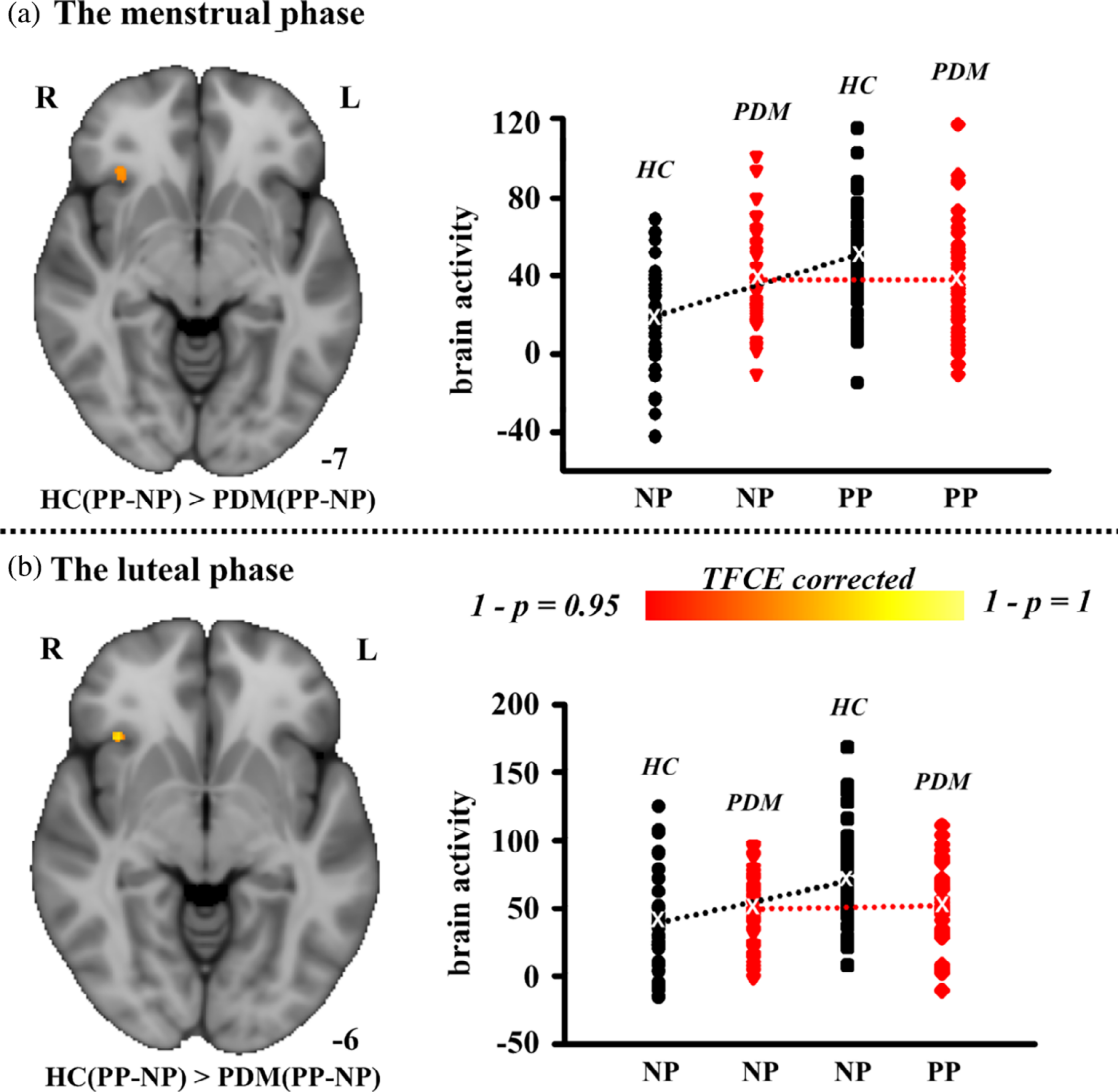
Between‐group differences in brain activation in response to visual stimuli. (a) Brain areas that were significantly more active in HC subjects than in PDM subjects while viewing pain pictures relative to neutral pictures (there were no regions that were significantly more active in PDM subjects than in HC subjects) during the menstrual phase. The average value for the contrast of parameter estimates, corresponding to brain activity in response to different types of visual stimuli of each subject, is plotted for the cluster representing the interaction effect (group × picture). (b) Brain areas that were significantly more active in HC subjects than in PDM subjects while viewing pain pictures relative to neutral pictures (there were no regions that were significantly more active in PDM subjects than in HC subjects) during the luteal phase. The average value for the contrast of parameter estimates, corresponding to brain activity in response to different types of visual stimuli of each subject, is plotted for the cluster representing the interaction effect (group × picture). A permutation test (permuted 5,000 times) with a TFCE was utilized for multiple comparison corrections. HC, healthy controls; PDM, women with primary dysmenorrhea; NP, neutral pictures; PP, pain pictures; L, left; R, right

Between‐group comparison (pain pictures ‐ neutral pictures) showed that only the right AI exhibited a significant interaction effect (group × picture) (*p* < .05, TFCE corrected, see Figure 4(a)). Post‐hoc analyses confirmed that, compared with the HC group, the right AI failed to be activated in PDM women when pain pictures were observed relative to neutral pictures (*p* < .05, TFCE corrected, see Table 2).

### 3.3 Between‐group differences in brain activity in response to visual stimuli during the luteal phase

The results for the luteal phase were similar to those for the menstrual phase. When viewing pain pictures, the main effect was significantly higher activation in the bilateral AI, MCC, ACC, and the left inferior parietal lobule (*p* < .05, TFCE corrected, see Figure 3(b) and Table 3). Additional activations were observed in the thalamus, inferior frontal gyrus, amygdala, precentral gyrus, and supramarginal gyrus (Table 3).

**TABLE 3:** Results of main effect and interaction effect during the luteal phase (*p < .05*, TFCE corrected based on the defined ROIs)

			Peak coordinate			
Region of activation	Side	Sizes	x	y	z	*t‐score*
The main effect of picture (PP vs NP)						
Insula	L	542	‐34	24	‐2	5.1695
Insula	R	173	30	20	‐2	4.1389
Thalamus	L	49	‐10	‐20	0	4.8614
Inferior frontal gyrus,opercular part	L	54	‐54	8	22	4.7792
Inferior parietal lobule	L	64	‐52	‐28	42	4.8702
Middle cingulate gyrus	L	135	‐6	18	34	5.2433
Anterior cingulate gyrus	L	44	‐7	22	30	4.2196
Amygdala	L	33	‐25	‐7	‐10	3.7975
Precuneus	L	104	‐55	7	19	4.5982
Supramarginal gyrus	L	159	‐53	‐27	37	4.5986
The interaction effect (HC [PP ‐ NP] vs PDM [PP ‐ NP])						
Insula	R	31	29	23	‐7	3.8594

*Note:* The ROIs is defined as empathy‐related and pain brain‐related regions from the meta‐analytical database of NeuroSynth. Peak coordinates refer to the MNI space.

Abbreviations: L, left hemisphere; MNI, Montreal Neurological Institute; NP, neutral pictures; PP, pain pictures; R, right hemisphere; ROIs, regions of interest.

Similarly, only the right AI exhibited a significant interaction effect during the luteal phase (*p* < .05, TFCE corrected, see Figure 4(b)).

### 3.4 Pain rating and brain activity within anterior insula were differently correlated between the two groups during the menstrual and luteal phases

**FIGURE 5:**
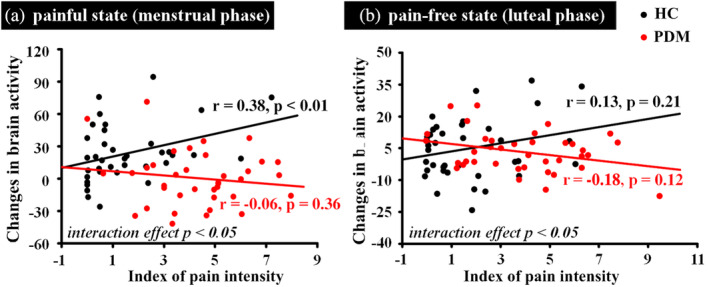
The Spearman's rho correlation between index of pain intensity and brain activity induced by visual stimuli. (a) During the menstrual phase, correlation between the change in hemodynamic signals induced by visual stimuli and the individual's index of pain intensity. (b) During the luteal phase, correlation between the change in hemodynamic signals induced by visual stimuli and the individual's index of pain intensity. HC, healthy controls, PDM, women with primary dysmenorrhea

Given the interesting results regarding index of pain intensity, between group differences in correlation between changes of AI activation and index of pain intensity were analyzed by using a regression analysis in the HC and PDM group. In our results, the right AI showed a significant interactive effect between different groups (*p < .05*). As shown in Figure 5(a), during the menstrual period, a positive correlation (*p < .01*, *r = .38*) was observed in the HC group; whereas no correlation (*p = .36*, *r = −.06*) was observed in the PDM group. During the luteal phase, similar correlation pattern was observed. As shown in Figure 5(b), a trend toward positive correlation was found for HC group (*p = .21, r = .13)*, while a trend toward negative correlation was found for PDM group (*p = .12, r = −.18*).

### 3.5 Between‐state differences in empathy ability and brain activity in response to visual stimuli

In this fMRI study, the ANOVA of the hemodynamic signal with repeated‐measures factors of state (painful, pain‐free) × group (HC, PDM) × visual stimuli (pain pictures, neutral pictures) did not yield a significant group × state × picture interaction effect, even after lowering the significance threshold to *p* < .001 (uncorrected).

## 4 DISCUSSION

In our results, the participants with PDM had abnormally higher pain empathy compared with healthy participants, during either the menstrual phase or luteal phase, and such abnormal pain empathy was found to be closely related to the brain activation of the AI, through use of task fMRI. It is important to note that there was no significant difference in pain empathy and brain activation between the menstrual and luteal phases. These findings suggest that repeated menstrual pain could affect the function of the central nervous system, thus causing changes in the perception of pain in others, regardless of the pain or pain‐free phases.

### 4.1 Brain activity in response to different types of visual stimuli during the pain phase

Long‐term menstrual pain not only induces chronic pelvic pain (Berkley, 2013; Lee et al., 2018), it may also lead to maladaptive neuroplasticity of the brain (Katy et al., 2011), which may further affect women's emotions, cognition, and their psychological modulation (V. A. Apkarian et al., 2004; Bajaj, Bajaj, Madsen, & Arendt‐Nielsen, 2002; Kim et al., 2014; Rhudy & Bartley, 2010; Tu et al., 2013; Von Deneen et al., 2019). Previously, Preis et al. (2013) reported that prior pain experience could lead to a higher level of empathy (Preis et al., 2013). Consistent with these studies, our study found that the PDM group had a significantly higher pain rating than the HC group when observing pain and neutral pictures, which indicates that women who are experiencing menstrual pain have a greater ability to sense the pain of others.

Neuroimaging studies have shown reproducible findings of a significant activation of the AI in healthy individuals in response to others' pain (Cui, Abdelgabar, Keysers, & Gazzola, 2015; Jackson et al., 2005; Lamm et al., 2011; Singer et al., 2004; Singer et al., 2006). Consistent with these studies, we also found a close relationship between AI activation and behavior response when viewing pain pictures relative to neutral pictures, in both the HC and PDM groups during their menstruation phase, which suggests a basis of affective shared neural representation during pain empathy (Jauniaux et al., 2019). However, compared with the HC group, no difference in brain responses in the right AI was found in PDM women when viewing pain pictures relative to neutral pictures. This disconnection between behavioral and neural responses may indicate an impairment of regulatory capacity of the right AI in pain empathy. It should be noted that we only found dysfunction of the AI in the two‐way ANOVA (group × picture) analysis, which was even lower than the threshold for statistical significance. Additionally, our results indicated a moderate correlation between changes of right AI activation and the rating differences between the picture types for the HC group, but there was none for the PDM group, which may further support our inference.

Various neuroimaging studies have shown that the insula may play an important role in pain information processing and modulating/receiving sensory input from visceral organs, as well as integrating sensory information with a pain effect (Dun et al., 2016; Li Hu & Iannetti, 2016; Katja et al., 2010; Low et al., 2018; Nieuwenhuys, 2012; L. Q. Uddin, Nomi, Hébertseropian, Ghaziri, & Boucher, 2017; Von Deneen et al., 2019). Morphological abnormalities and dysfunction of the insula have been widely found in many chronic pain conditions (Dun et al., 2016); for example, chronic back pain (Fritz et al., 2016), chronic complex regional pain syndrome (Geha et al., 2008), fibromyalgia (Yunus, 2007, 2008), irritable bowel syndrome (K. D. Davis et al., 2008) and tension headache (Schmidt‐Wilcke et al., 2005). A previous study of ours also found lower brain gray matter volume in the AI, and its association with the intensity of menstrual pain (Dun et al., 2016), which may indicate a significant role of the AI for the subjective experiencing of pain in PDM women. On the other hand, although it is suggested that the AI is an important brain region for pain empathy, cumulative evidence from other research fields has implicated a variety of other functions of the AI (Jauniaux et al., 2019). Insights from neuroimaging studies suggest that the AI is a key node of the salience network related to the brain function involved in detecting, orienting attention toward, and reacting to salient sensory events (Legrain, Iannetti, Plaghki, & Mouraux, 2011; Lucina Q Uddin, 2015). Hence, dysfunction of the AI may be related to complex and pervasive abnormalities in behaviors. In our study, we speculated that long‐term nociceptive information about visceral stimuli may affect the function of the affective component of the pain experience in the right AI, which may lead to the loss of the AI in detecting salient cues related to others' pain. Since most existing studies have shown the key role of the AI in pain empathy only in healthy subjects, there is less knowledge in clinical populations, so the specific underlying physiological changes contributing to our observation remain in question.

Recent neuroimaging studies have shown that somatosensory areas are also involved in the emotional response to pain, leading to an empathic response when watching others suffer pain in picture‐based paradigms (Lamm et al., 2011). Jauniaux et al. (2019) showed that mirroring mechanisms might account for the activation of an embodied somatosensorimotor representation of another person's pain during pain observation (Jauniaux et al., 2019). In our findings, we also found significant brain activation of sensorimotor regions when viewing pain pictures relative to neutral pictures. However, we did not find any significant interaction effect (group × picture) in the sensorimotor regions. Our imaging study on empathy for pain suggested that the sensory component of pain empathy in PDM women may be not affected by long‐term menstrual pain.

### 4.2 Comparison of brain activity in response to different types of visual stimuli between pain and pain‐free phases

In our results, when observing pictures and neutral pictures, the PDM group had a significantly higher pain rating than the HC group, even in the absence of menstrual pain. Neuroimaging studies of PDM have found rapid state‐related brain morphological and metabolic changes between the pain and pain‐free states (Cheng‐Hao et al., 2009; Tu et al., 2013; L. Yang et al., 2019). Several studies have also shown that dysmenorrhea is associated with abnormal trait‐related structural and functional changes during the pain‐free phase (Katy et al., 2011; Tu et al., 2010). Our results indicated decreased right AI activation and increased pain rating when observing pain pictures relative to neutral pictures during the pain‐free phase, which suggests that the effect of long‐term cyclical pain on empathy may not only be reflected in the pain phase but also extend to the pain‐free phase. Interestingly, there was no significant difference in brain activation and pain rating for the comparison between pain and pain‐free phases. This indicates that a high level of empathy associated with long‐term pain in PDM did not vary with the pain or pain‐free state across the menstrual cycle.

Previous studies have revealed that empathy can be sub‐classified as either trait empathy or state empathy (Eisenberg, Eggum, & Di Giunta, 2010; Eisenberg & Strayer, 1990; Wood, James, & Ciardha, 2014; Y. Yang et al., 2017). Trait empathy refers to relatively stable psychological characteristics, which include the ability of individuals to resonate with others' emotions and emotional states (Eisenberg & Strayer, 1990; Y. Yang et al., 2017). State empathy is a psychological state triggered by the corresponding situation, which mainly emphasizes the empathic response generated during the interaction between individuals and the environment (Eisenberg & Strayer, 1990; Y. Yang et al., 2017). It has been suggested that rapid adaptive and maladaptive changes in the brain may occur simultaneously during the switching from menstrual pain to the pain‐free phase in dysmenorrhea women (David, Nasim, Lino, & Bruce, 2012; Tu et al., 2013). In this present study, the cumulative maladaptive effects resulting from repetitive rapid plasticity in the brain, in conjunction with the early onset of dysmenorrhea, may generate more psychological distress and negative emotions in these women than in nondysmenorrhea women (David et al., 2012; Tu et al., 2013). It is conceivable that the aberrant pain empathy in PDM, whether in the pain or pain‐free phase, may be a stable psychological trait caused by the maladaptive plasticity of the brain function, which does not change with the specific menstrual phases of the participant. These findings give important and novel insights into the effects that maladaptive changes in the brains of PDM women have on empathy for pain, and also provide some references for the study of maladaptive plasticity related to physical and psychological problems in other pain disorders.

### 4.3 Effect of menstrual pain on the insula and on pain empathy

David et al. (2012) proposed an allostatic load model to understand the effect of frequent behavioral or physiological stressors on body function (David et al., 2012). They pointed out that the individual may undergo an adaptive process at the beginning of the stressor, and that the central and peripheral physiology and function can generate maladaptive changes with the accumulation and persistence of the stressor (David et al., 2012). A previous study on cyclic menstrual pain reported a maladaptive reorganization of gray matter volumes in brain regions related to pain information processing and modulation (including somatosensory cortex, insula, and cingulate cortex) (Low et al., 2018). These structural changes might constitute the architecture that underlies altered corresponding cortical function (Laneri et al., 2017). It is important to note that the bilateral AI and ACC were regarded as shared neural substrates for the actual experience of pain and empathy elicited by the pain of others (Lamm et al., 2011; Preis et al., 2013; Singer et al., 2004). This finding suggests that recurrent experiencing of dysmenorrheic pain in the central nervous system indirectly affects empathy by altering activity in the insula.

## CONFLICT OF INTERESTS

The authors declare no potential conflict of interest.
